# Trend Analysis of Visceral Leishmaniasis at Addis Zemen Health Center, Northwest Ethiopia

**DOI:** 10.1155/2014/545393

**Published:** 2014-03-25

**Authors:** Yitayih Wondimeneh, Yegnasew Takele, Asmamaw Atnafu, Getachew Ferede, Dagnachew Muluye

**Affiliations:** ^1^School of Biomedical and Laboratory Sciences, College of Medicine and Health Sciences, University of Gondar, P.O. Box 196, Gondar, Ethiopia; ^2^Gondar University Leishmaniasis Research and Treatment Centre, University of Gondar, P.O. Box 196, Gondar, Ethiopia

## Abstract

*Background.* Visceral leishmaniasis (VL) is a systemic disease caused by the *Leishmania donovani* complex. It is one of the fatal diseases if left untreated. In Ethiopia, there are many VL endemic foci. The aim of this study was to determine the trends of VL in the study area. *Methodology.* A retrospective study was conducted at Addis Zemen health center from September 2005 to August 2011. Data were collected from laboratory registration book and entered and analyzed by using SPSS version 20 software and *P* value of ≤0.05 was considered statistically significant. *Result.* A total of 7161 VL suspected cases were reported in the study area. The overall prevalence of VL was 2801 (39.1%). Of the 2801 VL positive cases, the highest annual prevalence, 988 (46.8%), was reported in 2005 but the trend gradually decreases. Majority of the VL confirmed cases were in the age groups of 5–14 years and males were more affected. *Conclusion.* The prevalence of VL in the study area was high in early 2005 but, gradually, the trend has been decreased and it becomes one of VL endemic foci in Ethiopia.

## 1. Background

Visceral leishmaniasis (VL) is a systemic disease caused by the* Leishmania donovani* complex and it is fatal if left untreated [1]. An estimated 500,000 new cases of VL occur annually [2]. More than 90% of VL cases occur in six countries: Bangladesh, India, Nepal, Sudan, Ethiopia, and Brazil. Migration, lack of control measures, and HIV-VL coinfection are the three main factors driving the increased incidence of VL [2, 3]. Other factors associated with increased mortality include HIV infection, severe malnutrition, pneumonia, and tuberculosis [4].

In east Africa, the responsible parasite for VL is* L. donovani* and the predominant mode of transmission is via sandflies biting and it is anthroponotic. Humans with VL or post-kala-azar dermal leishmaniasis provide the major reservoir for transmission; thus, incomplete or irregular treatment of VL leads to drug pressure and parasite resistance [5]. Incident asymptomatic* L. donovani* infection in VL high-endemic foci countries is nine times more frequent than incident symptomatic VL disease. About 1 in 50 of these new but latent infections led to VL within the next 18 months [6]. An effective life-long cellular immune response normally develops, and residual parasites are suppressed unless immunodeficiency is present [7].

Though there are high mortality and prevalence rates throughout the world, the magnitude of the problem is still underestimated due to different factors [8, 9]. For example, in India VL prevalence and mortality is high but underreporting is a big problem due to communication barriers between the private health institution that handle many VL cases and the Indian Ministry of Health [10].

In Ethiopia, especially in the northwestern part of the country, there are many VL foci. According to the Ethiopian ministry of health estimates, the annual burden of VL is expected to be between 4,500 and 5,000 cases [11]. Despite this fact there is no specific data showing the trend prevalence of VL in the study area. Knowing the magnitude of the problem at a different time and situation is very important for the development of prevention and control strategies. This study attempts to determine the seven years trend prevalence of VL in Adis Zemen health center, Northwest Ethiopia.

## 2. Methodology

### 2.1. Study Design, Area, and Period

**Figure 1:**
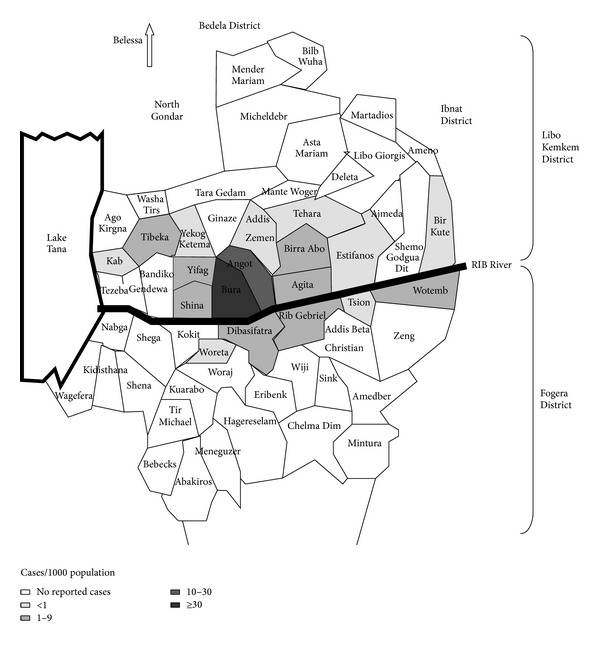
Physical map of the study area and nearby districts: Alvar et al. 2007 [18].

A retrospective study was conducted at Addis Zemen health center from September 2005–August 2011. Addis Zemen is found in South Gondar administration zone in the Amhara region of northwestern Ethiopia and is around 637 km far from the capital city of Ethiopia. Addis Zemen is the capital town of Libo Kemkem wereda (district), which has average populations of 198,374. It has an average altitude of 2,000 m above sea level. The health center serves not only Libo Kemkem district but also the nearby districts like Fogera (which has estimated populations of 226,595) (Figure 1).

### 2.2. Source Population and Study Participants

The source populations were all the Libo Kemkem and the nearby districts people who have the access to be served at Addis Zemen health center. The study participants were all patients who have been suspected for visceral leishmaniasis infection and tested for direct agglutination test (DAT) at the time of their visit.

### 2.3. Sample Size and Sampling Procedures

By using a convenience sampling technique, we reviewed a laboratory registration book which contains VL direct agglutination test (DAT) results with a cut of value 1 : 1600 from 2005 to 2011. Data were collected manually from the registration book by using temporary work sheet which contains the required information. Finally, we have got a total of 7,161 VL cases during the study period.

### 2.4. Data Analysis

Data were checked for completeness, cleaned manually, and entered and analyzed using SPSS version 20 statistical package. The results were summarized by using frequency table and graph. Pearson's *χ* ^2^ test with 95% CI is computed as measures of association and *P* value of ≤0.05 was considered as statistically significant.

### 2.5. Ethical Considerations

Ethical clearance was obtained from University of Gondar Research and Community Service Core Processor Ethical Committee. Supportive letter was also obtained from College of Medicine and Health Sciences. We have explained the purpose and importance of this research to the responsible official person. After getting permission, all the required information and laboratory results of VL were collected.

## 3. Result

From 2005 to 2011, a total of 7161 VL suspected cases were reported at Addis Zemen health center. Of all, 5,155 (72.0%) were males (with the mean age of 23 ± 14) and 2006 (28.0%) were females (with the mean age of 21 ± 14). The age of the study participants ranges from 1 to 85 years. The median age of the study participants was 20 year. The majority of the study participants (38.9%) were in the age groups of 15–29 years and 92.8% of the VL suspected cases were from rural areas (Table 1).

During the study period, a total of 7,161 VL cases were suspected and requested for laboratory analysis. Direct agglutination test (DAT) was performed to confirm the presence of VL infection at a time of patient visit. Out of the total VL suspected cases, 2801 (39.1%) were positive. The highest (50.1%) convenience sample prevalence of VL was found in the age groups of 5–14 years. Males were more affected than females (40.1% versus 36.5%) (*P* = 0.005). Individuals who live in the rural area were more affected by VL than those who live in urban areas (39.5% versus 34.4%) (*P* = 0.022) (Table 2).

**Figure 2:**
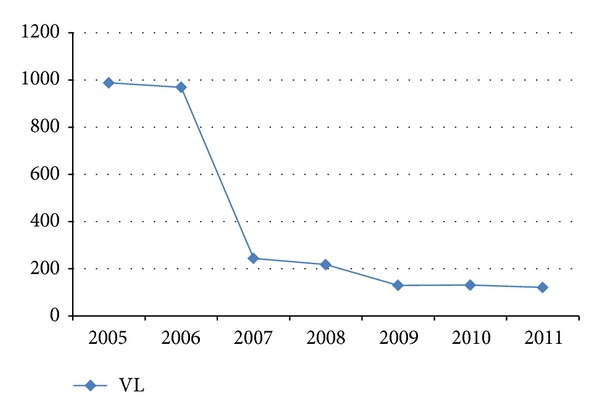
DAT positive results from 2005 to 2011.

There was a fluctuating trend of VL within the last seven years and the maximum, 988 (46.8%), laboratory confirmed cases of VL were reported in 2005, but, gradually, the trend has been decreased (Figure 2).

## 4. Discussion

Visceral leishmaniasis is a huge public health problem in terms of morbidity and mortality [3]. It is also a big burden of health care facilities throughout the world including Ethiopia. The disease is spreading and the new endemic foci are now being reported in different countries including the study area [12].

In the present study, the overall convenience sample prevalence of VL was 2801 (39.1%). The majority (50.1%) of the VL confirmed cases were in the age groups of 5–14 years. This might be associated with their daily activities. In the study area, these young study participants are expected to keep domestic animals outdoors especially during dawns. This may have exposed them to the bite of sandflies. Males were more affected than females (40.1% versus 36.5%). A similar finding was also reported in other studies [13–16]. This gender difference might be due to differences in outdoor activities between males and females. As indicated in another study [13], males are more involved in outdoor activities than females in the study area and this may have exposed them more to the bites of sandflies [16].

In this study, majority, 2624 (39.5%), of the VL confirmed cases were from rural settings. This may be associated with sandfly bride sites and it is assumed that the breeding sites are found more concentrated in the rural areas than in urban environments [13]. However, in the present study, there were also cases in the urban area which could be attributed to traveling to endemic areas [17]. Moreover, it has been reported that cases reported in urban areas are mostly due to traveling to endemic areas [13].

From the total VL cases, the highest convenience sample prevalence of VL cases was reported in 2005: 988 (46.8%), and 2006: 969 (38.5%). This was a time where there was an outbreak of VL in Libo Kemkem and its surroundings [18]. In the previous survey, environmental factors, host factors, and labor migrants to the endemic areas were reported as a factor contributing to this epidemic [4, 13, 18]. However, the number of cases reported from 2005 to 2006 has dropped dramatically and this might be due to early diagnosis and treatment of VL cases in 2005 and 2006 because of the outbreak. There are some reports in other countries that indicate early diagnosis and treatment that can help in controlling VL outbreaks and transmission [19, 20].

There was also a dramatic decrease in the number of VL cases detected from 2007 to 2011 and it remains low or nearly stationary due to early diagnosis and treatment of VL epidemic cases. Nowadays, the study area is considered as VL endemic environment due to various factors like population migration to and from other endemic areas, malnutrition, and HIV/AIDS [2, 3, 11]. Hence, complete eradication is a big challenge unless integrated prevention and control mechanism are applied.

## 5. Conclusion

The prevalence of VL in the study area was high in early 2005 and it was considered as epidemic but, gradually, the trend has been decreased and it becomes one of VL endemic foci in Ethiopia. Males were more affected by VL diseases due to their outdoor activity. To minimize the disease burden, we have to increase the community awareness about the diseases prevention and control strategies by giving health education using health extension workers, minimizing dawns outdoor activities, early diagnosis and treatment of known cases, and reducing migration to VL endemic foci.

## Figures and Tables

**Table 1:** Sociodemographic characteristics of the study participants at Addis Zemen health center, northwest Ethiopia, 2005–2011.

Characteristics	Frequency	Percent (%)
Age group		
<5	761	10.6
5–14	1462	20.5
15–29	2789	38.9
30–44	1568	21.9
≥45	581	8.1
Gender		
Male	5155	72.0
Female	2006	28.0
Residence		
Rural	6646	92.8
Urban	515	7.2

**Table 2:** Sociodemographic characteristics of the study participants in relation to VL at Addis Zemen health center, northwest Ethiopia, 2005–2011.

Characteristics	VL		*P* value
	Negative *N* (%)	Positive *N* (%)	
Age groups			
<5	383 (50.3)	378 (49.7)	<0.001
5–14	730 (49.9)	732 (50.1)	
15–29	1727 (61.9)	1062 (38.1)	
30–44	1120 (71.4)	448 (28.6)	
≥45	400 (68.8)	181 (31.2)	
Gender			
Male	3087 (59.9)	2068 (40.1)	0.005
Female	1273 (63.5)	733 (36.5)	
Residence			
Rural	4022 (60.5)	2624 (39.5)	0.022
Urban	338 (65.6)	177 (34.4)	

## Limitation of the Study

Due to the nature of the study, retrospective analysis and convenience sampling technique, we were unable to get all the differential diagnosis, drug regimens, and treatment outcomes from the registration book. In addition, this study was carried out in the health institution and the result of this study may not infer to the general population. Furthermore, this study only depends on serological tests. There is no microscopically confirmed parasitological data in different seasons in a given year. To fill all these gaps, there is a need for further study with different study design.
